# 1167. Changing spectrum of aetiology of Acute Fever with Jaundice (Tropical Jaundice) in Adults presenting to Emergency Care--- A study from a Tertiary Care Hospital in North India

**DOI:** 10.1093/ofid/ofac492.1004

**Published:** 2022-12-15

**Authors:** Vikas Suri, Mandeep Singh, Ajay Duseja, Manisha Biswal, M P Singh, Kapil Goyal, Ritin Mohindra, Harpreet Singh, Ashish Bhalla, R Ratho

**Affiliations:** PGIMER, CHANDIGARH, INDIA, Chandigarh, Chandigarh, India; PGIMER, CHANDIGARH, Chandigarh, Chandigarh, India; PGIMER, Chandigarh, Chandigarh, India; PGIMER, Chandigarh, Chandigarh, India; PGIMER, Chandigarh, Chandigarh, India; PGIMER, Chandigarh, Chandigarh, India; PGIMER, Chandigarh, Chandigarh, India; PGIMER, Chandigarh, Chandigarh, India; PGIMER, Chandigarh, Chandigarh, India; PGIMER, Chandigarh, Chandigarh, India

## Abstract

**Background:**

The spectrum of infections causing the above acute onset fever with jaundice has changed over the last few years from the conventional hepatotropic viruses to new emerging infections.

**Methods:**

250 adult patients ≥ 14 years of age) with acute fever (body temperature > 101ᵒF of 14 days or less in duration) without any localized source of infection on initial clinical evaluation accompanied with jaundice (hyperbilirubinemia ≥ 1.5 mg/dl or elevation of ALT or AST ≥ three times upper limit) were enrolled.All these patients with fever and jaundice were evaluated on the basis of a standard proforma and were evaluated for malaria (peripheral smears/rapid diagnostic kits), scrub typhus( PCR /IgM ELISA), leptospirosis(IgM ELISA), enteric fever by blood cultures and dengue by dengue (NS1 antigen test and IgM ELISA), Hepatitis(IgM ELISA of EBV/HSV, IgM ELISA of HAV/HEV and HBsAg with IgM HBc ELISA if HBsAg positive)

**Results:**

62.5 % were males and 37.5 % were females. The mean duration of fever before the presentation was 8.1 ± 2.58 days. 10 patients (4%) died, while 133 patients (96%) improved with treatment. Scrub typhus 57 (22.8%), Hepatitis E 33 (13.2%), malaria 9 (3.6%), dengue fever, enteric fever, hepatitis A and leptospirosis in 26 (10.4%), 6 (2.4%), 6 (2.4%) patients and 4 (1.6%) patient respectively were the prominent aetiology a patient presenting with fever and jaundice. Probable sepsis (Fulfilling SIRS criteria with a negative culture) accounted for 40(16%) patients. In 69(27.6%) cases no diagnosis could be made on serological testing Conjunctival suffusion (OR=23.17), respiratory crepitations (OR=5.17), thrombocytopenia (OR=1.14), normal INR (OR=0.29) were significant predictors of a diagnosis of scrub typhus in patients with fever and jaundice. Severe anaemia (Hb< 8), Hypoalbuminemia, severe thrombocytopenia (Platelet count < 50,000) and a near-normal INR at admission were predictors of a malarial vs a viral aetiology of Tropical jaundice. Co-infection with scrub typhus and malaria was seen in 6 patients (vivax-5 and falciparum-1) and viral hepatitis A & E was observed in 10 patients.

**Conclusion:**

Neglected Tropical diseases like Scrub typhus infection is emerging as common aetiology of acute onset tropical jaundice in adults presenting to emergency services.

**Disclosures:**

**All Authors**: No reported disclosures.

